# High resolution multi-facies realizations of sedimentary reservoir and aquifer analogs

**DOI:** 10.1038/sdata.2015.33

**Published:** 2015-07-07

**Authors:** Peter Bayer, Alessandro Comunian, Dominik Höyng, Gregoire Mariethoz

**Affiliations:** 1 Department of Earth Sciences, ETH Zurich, Zurich 8092, Switzerland; 2 Dipartimento di Scienze della Terra ‘A.Desio’, Università degli Studi di Milano, Milan 20129, Italy; 3 Center for Applied Geosciences, University of Tübingen, Tügingen 72074, Germany; 4 Institute of Earth Surface Dynamics, University of Lausanne, Lausanne 1015, Switzerland

**Keywords:** Hydrogeology, Sedimentology

## Abstract

Geological structures are by nature inaccessible to direct observation. This can cause difficulties in applications where a spatially explicit representation of such structures is required, in particular when modelling fluid migration in geological formations. An increasing trend in recent years has been to use analogs to palliate this lack of knowledge, i.e., exploiting the spatial information from sites where the geology is accessible (outcrops, quarry sites) and transferring the observed properties to a study site deemed geologically similar. While this approach is appealing, it is difficult to put in place because of the lack of access to well-documented analog data. In this paper we present comprehensive analog data sets which characterize sedimentary structures from important groundwater hosting formations in Germany and Brazil. Multiple 2-D outcrop faces are described in terms of hydraulic, thermal and chemical properties and interpolated in 3-D using stochastic techniques. These unique data sets can be used by the wider community to implement analog approaches for characterizing reservoir and aquifer formations.

## Background & Summary

Sedimentary reservoirs exhibit a rich diversity of composition and topology of internal structures, with physical and chemical properties varying significantly in space. As a result, the description of flow and transport processes can be challenging, affecting subsequent understanding of groundwater contaminant transport, geothermal circulations, hydrocarbon migration in reservoirs or carbon storage and sequestration processes. To date, highly parameterized numerical models are the most general tools to gain insights into processes taking place in the subsurface^1,2^. Often a limited number of boreholes are the only direct observations, and the space between these sparse measurements needs to be filled by interpolation. Additional information is sometimes available from hydrogeological, hydrochemical and geophysical field investigation, but the resolution of such field measurements rarely reaches that of numerical models. For instance, contaminant transport processes in aquifers generally require a resolution at the sub-meter scale, which is not accomplished by standard field testing^3–5^. Since transport models are most influenced by geological heterogeneity, they inevitably carry substantial uncertainty.

Natural analogs have proven invaluable to palliate the lack of data prevalent in many cases^6–12^. The underlying principle is simple: we learn from exposed geological formations to describe hidden ones, assuming that subsurface system and analog share similar properties. More specifically, the geological patterns learned from analogs can be used to constrain geostatistical models of spatial continuity^13–16^. At the same time, detailed analog models facilitate surrogate analysis of specific flow and transport processes^17–20^ and enable cost-effective testing of new field techniques in virtual but realistic systems^21–23^. Despite the appeal of the analog approach, it is not widely used in geological or hydrogeological modelling. The main reason is the lack of free and publicly accessible databases^2,24^. Creating digitized high resolution characterizations by detailed mapping of archetypal outcrops is time consuming^25^. Moreover, such mapping only provides two-dimensional (2-D) analogs, while flow and transport modelling often requires three-dimensional (3-D) characterization. Therefore, it can be difficult to find a representative 3-D analog for a given site. Ideally a freely accessible repository of type locations would exist, spanning a broad range of characteristic subsurface environments. Here, we take a first step in this direction.

Solutions that have been proposed to develop 3-D analogs include the combination of point measurements, outcrop data and geophysical surveys^7,26^. Additionally, strong research efforts have been dedicated to developing advanced geostatistical techniques that are able to extract information from spatially distributed and incomplete data^15,27,28^. Such methods can be used to generate unconditional realizations of spatially distributed geological parameter fields, which serve as realistic but location-independent representations of the subsurface. Ensembles of multiple such realizations can be used for quantifying uncertainty when modelling a given sedimentary system^29,30^.

In this work, we present multiple realizations of two different sedimentary analogs. We chose a moderately heterogeneous fluvial-aeolian deposit of the upper part of the Pirambóia Formation (Triassic) of south-eastern Brazil^31^, and a highly heterogeneous fluvio-glacial braided river sediment from the Pleistocene in the upper Rhine valley of southern Germany^32^. These analogs were mapped independently, but by following a similar procedure: In order to obtain 3-D images, multiple outcrop cross sections were sequentially mapped and digitized during ongoing excavation in gravel pits.

We focus on a range of different geological, physical and chemical properties, with the purpose of developing genuine portrayals of the selected sedimentary blocks at the sub-decimetre scale. These properties are associated with sets of facies types specific to each analog. Such facies represent the smallest homogenized units and therefore do not represent up-scaled values. Mapping their distribution on each outcrop results in a digitized facies mosaic. These mosaics allow for visualizing the sedimentary structures in the vertical outcrops, and the 3-D spatial changes are captured by combining all mapped cross-sections at a site. By sharing multiple realizations of both analogs, we open the door for any kind of stochastic flow and transport modelling in those types of geological environments. The analogs can serve as benchmarks for different levels of hydraulic, hydrochemical and thermal heterogeneity.

## Methods

### Definition of facies types

Four different facies types are distinguished in the analogs. These address sedimentological, hydrogeological, chemical and geothermal criteria:

- *Lithofacies*: represents a subdivision of a stratigraphical unit, which stems from a distinct deposition event or environment^11,33^.
- *Hydrofacies*: denotes a quasi-homogeneous unit that can be described by characteristic hydraulic properties^33,34^.
- *Chemofacies*: classifies units with same chemical attributes^35–37^.
- *Thermofacies*: subdivides units according to homogeneous or homogenized thermal properties^38,39^.

The facies types represent mappable clusters of the selected gravel bodies, at a minimum resolution of half a decimetre. They are determined based on a hierarchical approach: initially, lithofacies were predefined after sedimentological analysis at the site, including outcrops in the vicinity of the same depositional environments^11^. The lithofacies represent the basic categories. These are further distinguished for defining hydrofacies units, when hydraulic properties reveal significant internal variability. This is especially the case when sediment grains show grading, or when a predefined lithofacies assembles small-scale interbedded strata. The hydrofacies-based classification is not further subdivided. This means chemical and thermal properties are estimated for the hydrofacies classes, which yields specific chemo- and thermofacies.

This hierarchical approach is chosen for several reasons. First, the different facies properties are often correlated. For instance, lithologically typical grain sizes result in similar hydraulic and thermal properties, and lithocomponents specific to a lithological strata often share similar chemical properties^40,41^. Second, consistent classification of different facies types simplifies their combined implementation in numerical models. Third, a crucial argument is the practicability of mapping, which is much easier when hierarchical classes are distinguished. This applies especially to field sampling and laboratory measurements^31^. Finally, qualitative classification guided by principal lithofacies types may lead to different hydro-, chemo- or thermofacies of sometimes similar quantitative properties. Therefore the presented classification offers the highest possible resolution, and depending on the model application facies types characterized by similar parameter values may be merged.

### Field and laboratory work

**Figure 1:**
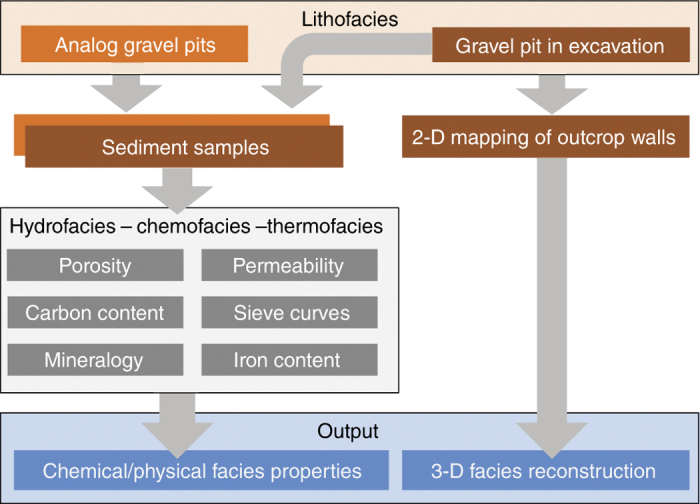
Workflow from field data collection to generation of three-dimensional aquifer analog realizations with litho-, hydro-, chemo- and thermofacies.

**Figure 2:**
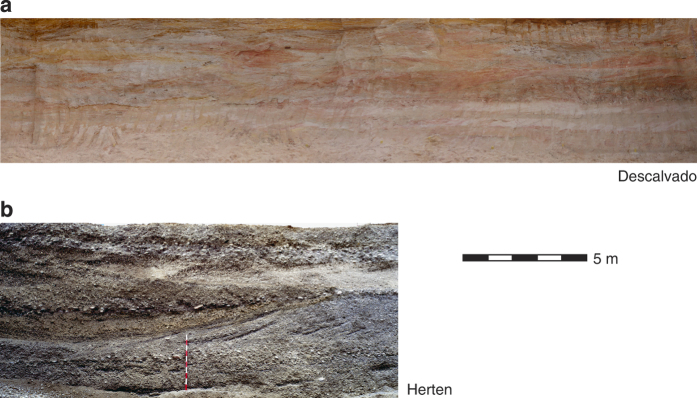
Outcrop photographs of Descalvado (**a**) and Herten (**b**) profiles taken at the gravel pits during excavation.

Both analog data sets are reconstructed rectangular blocks of unconsolidated sediments, which are mapped by several (5–6) outcrop cross sections in the field and supported by laboratory measurements (Fig. 1). Each data gathering campaign took around six months, synchronized with the mining activities in the sand and gravel pits. The fluvial-glacial sediments of the Rhine valley were excavated close to the town of Herten (Herten-analog) in the summer of 1999^11,32^, and the fluvio-aolian deposits in Brazil close to the town of Descalvado (Descalvado-analog) in 2011^31^ (Table 1). Outcrop photographs are shown in Fig. 2. Both case studies were mainly motivated from hydrogeology: the young Rhine gravels host among the most productive aquifers in central Europe, and the Brazilian Pirambóia formation belongs to those sequences that store the most important groundwater reservoir in South America, the Guarani Aquifer system (GAS). The original descriptions of highly resolved hydraulic and hydrochemical heterogeneity and earlier interpolated blocks hence served as aquifer analogs in several previous groundwater modelling and model inversion studies^17,19,20,22,28,42,43^. For instance, Maji and Sudicky^42^ implemented the Herten analog in a numerical flow and transport model with decimetre-grid resolution to examine the solution of dense nonaqueous phase liquids (DNAPL) in sedimentary aquifers. Werth, *et al.*^17^ demonstrated with one Herten cross section, how contaminant mixing and flow focusing is controlled by typical sedimentary structures. Kowalsky, *et al.*^23^, Jiménez, *et al.*^43^ and Hu, *et al.*^22^ chose Herten profiles for developing and validating new geophysical and tomographical field investigation techniques. In these studies, the analog served to set up a realistic but computer-based virtual aquifer. In comparison to experiments in the field, computer experiments are less costly and, more importantly, can be validated using a reference where all hydraulic, thermal or chemical details are known. Our work offers new 3-D realizations of the Herten-analog, for the first time 3-D interpolations of the Descalvado-analog and a combined resolution of four different facies types. To our knowledge, there are currently no analog datasets publicly available with comparable multiple parameter characterizations of such sediment bodies.

The field and laboratory work for the Herten analog is described in detail in Bayer, *et al.*^32^ In total, six parallel and equidistant cross sections of 16 m×7 m were mapped at 2 m steps. The lithofacies classification follows the suggestions by Heinz and Aigner^44^, and four different categories are distinguished (Table 2) according to dominant grain size, sorting and texture. For systematic grouping, abbreviations denoting the structural and textural properties such as major grain sizes (e.g., ‘G’ for gravely and ‘S’ for sand) were chosen. Two of the lithofacies classes (well sorted gravel GS-x, well sorted sand S-x) are equivalent to hydrofacies categories, reflecting that hydraulic properties show only minor variations within the lithologically defined units. This is not the case for the alternating gravel, as well as for the poorly sorted, matrix supported gravel lithofacies. For the latter, a cobble-rich and a sand-rich hydrofacies is subdivided. The alternating gravel lithofacies shows great internal variations in the hydraulic properties, and thus it is split up into five different hydrofacies categories. These delineate interchanging, typically cross-bedded gravel sequences. They are among the most striking features in the cross sections, with small-scale variations and interbedded highly permeable open framework gravels (cGcg,o, sGcg,o). Bayer, *et al.*^32^ also distinguish six up-scaled architectural layers (or wedges) to combine lithofacies of the same depositional regimes. The alternating sequences mainly form the central share of the block, whereas continuous and relatively regular layers of gravel represent the top and bottom parts.

Höyng, *et al.*^31^ present a comprehensive report of the field campaigns, the laboratory measurements and the derived cross sections of the Descalvado-analog. The lateral width of the examined outcrop walls is 28 m and thus longer than that for the Herten-analog, given a similar vertical size (Table 1). Only three parallel profiles were mapped, however, at greater spacing of 3.5 m. These profiles are complemented by the two perpendicular lateral faces, which deliver a true 3-D picture of the structure. A fence diagram with the orientation of all five profiles is given by Höyng, *et al.*^31^, but no interpolated 3-D models have so far been constructed. Equivalent to the Herten case, five lithofacies are distinguished, four of these are sand dominated and one represents clay intraclasts (Table 3). Three of these lithofacies are further subdivided due to internal variability of grain sizes, and thus in total nine different hydrofacies are derived. In the entire mapped sediment block, three major architectural elements can be distinguished: the quasi homogeneous basis with well sorted medium to very fine aeolian sands (Sp), the more heterogeneous central part with cross-bedded coarse sand and gravel (SGt), and on top a laterally continuous layer of trough cross-bedded fine to medium sand facies (St). So in both analogs, highest variability is found in the centre.

For the distinction and characterization of the different hydrofacies types, hydraulic conductivity and porosity values were determined using undisturbed samples collected in the field. The porosity was derived by direct measurement in the laboratory, using multiple samples of each hydrofacies. In order to obtain hydraulic conductivity values, different methods have been used at both sites. Directly measured laboratory values are considered most reliable. At the Herten site, the hydraulic conductivities were determined as mean values of repeated flowmeter measurements. The samples of the ten different Herten facies were collected at adjacent gravel pits, with outcrops from similar fluvial deposits of glacial origin from the alpine region (Triassic, Jurassic, Creataceous and Tertiary rock formations). For the Descalvado, only the Fm facies was examined by laboratory permeameter testing. When no directly measured laboratory value is available, empirical estimates are chosen. For each individual facies, repeated sieving of multiple samples combined with a laser diffraction method was applied to derive grain size distributions. These were utilized in the empirical Kozeny-Carman, Beyer, Panda and Lake and USBR formula^11,31,32,45^ to estimate specific hydraulic conductivity values. The methods used for each individual hydrofacies are reported in detail in Bayer, *et al.*^32^ and Höyng, *et al.*^31^

Chemical heterogeneity is rarely reported for analogs, and it may be used to describe a broad range of variable chemical characteristics such as mineral composition, carbon content, etc. The selected chemofacies types of both analogs refer to different properties. At Herten, it is the organic carbon content, which is a main determinant of sorption capacity. For the Descalvado, which represents a more mature and quasi carbon free sediment, the iron content (Fe(III)) was examined. Sediment bound iron is abundant in most sediments, and Fe(III) is relevant, for instance, as a solid phase electron acceptor during degradation of organic contaminants in aquifers.

For the Herten facies types, in great detail the specific mineral and organic carbon content for given grain size ranges were determined^40,45^. Carbon contents are available only for four facies (Gcm, Gcm,b, Gcg,o, S-x), whereas sieve curves were measured for all ten. In order to extrapolate to the other facies, the carbon content values from the most similar measured lithofacies were adopted, that is, data from Gcm was used for cGcm and sGcm, data from S-x for GS-x, data from Gcm,b for fGcm,b, and data from Gcg,o for cGcg,o, sGcg,o. Summing up the grain size-specific carbon allows obtaining the total organic carbon mass fraction (*f*_oc_, mg/g) as a parameter characterizing chemofacies. Since no carbon measurements were carried out at the Herten site, we list in Table 2 those from two related sites: *f*_oc,S_ is based on an outcrop close to the town of Singen and *f*_oc,H_ from one located in the vicinity of the village of Hüntwangen. The carbon content of the latter shows only little variability, whereas *f*_oc,S_ spans a broader range. Since the same categories are used as for the hydrofacies, differences between some chemofacies are not significant, and thus these may be pooled in four or six major classes.

The Fe(III) content of the nine different Descalvado facies types was determined by laboratory testing of three samples per facies. For this, 0.5 g of sediment was filled in 58 ml serum bottles (triplicate of each sample). After adding 25 ml 0.5 M HCl, the samples were put on a shaker table for 1 h to dissolve amorphous and poorly crystalline Fe phases. Crystalline Fe was extracted by adding 6 M HCl to the sample and incubation for 24 h in a 70 °C water bath. The dissolved Fe(II) and Fe(III) were determined in the liquid phase by ferrozine assay. The purple-coloured ferrozine complex was quantified spectrophometrically at 562 nm using a microtiter plate reader (FlashScan 550; Analytik Jena, Jena, Germany). The concentrations of Fe(III) were determined by the difference between Fe(II) and Fe(total). Amorphous and poorly crystalline Fe phases could not be quantified because concentrations did not reach measurable levels (>10 μM). The presented solid Fe(III) oxides refer to highly crystalline phases (i.e., goethite).

The thermofacies are characterized by thermal conductivity (*K*_T_, W/mK) and specific heat capacity (*c*_P_, MJ/m^3^ K). These parameters are estimated indirectly based on the volumetric fractions of mineral components and porosity. The Herten facies mineral components (Quarz, Feldspar, Calcite) are determined with the same samples as used for the chemofacies characterization^40,45^. For these minerals, tabularized thermal properties are available^46^. Assuming water saturated conditions, the bulk thermal facies properties can be approximated by the geometric mean (*K*_T_) and the arithmetic mean (*c*_P_) of the individual components^46,47^. The same indirect method was used for the Descalvado case. The more mature sediment of this analog is assumed to be dominated by quartz. In the field, only minor local occurrence of calcite was found. As a rough approximation for the quartz arenitic facies^48^ of the Descalvado analog, the feldspar content is assumed to be 1/10 of that of quartz, which is a ratio reported for similar sediments^46^.

### Geostatistical modelling

**Figure 3:**
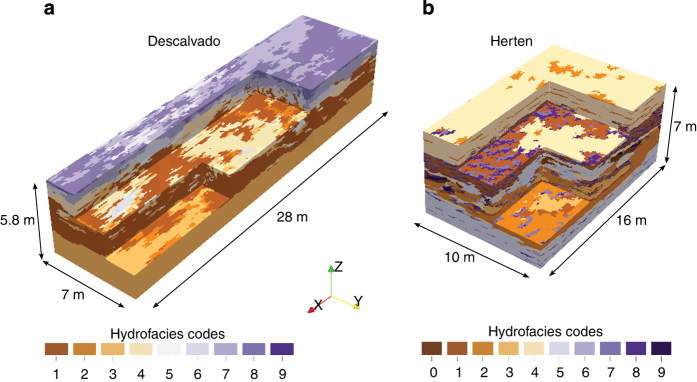
Visualization of two exemplary realizations for Descalvado and Herten aquifer analogs with colour coding of the facies types (see Tables 2 and 3).

Ensembles of multiple 3-D realizations of the Herten and the Descalvado analogs (Fig. 3) are obtained by multiple-point statistics (MPS) simulation^49,50^. MPS is a collection of geostatistical simulation tools that are specifically aimed at representing complex connected structures and curvilinear patterns. To apply MPS, a training image is normally required, i.e., a conceptual model of heterogeneity that has the same dimensionality as the domain to be modelled. For example, to simulate a 3-D domain with MPS, one normally needs a full 3-D training image. Comunian, *et al.*^51^ proposed a technique, named sequential 2-D simulation with conditioning data (s2Dcd), which relaxes the aforementioned requirement and allows the MPS simulation of 3-D domains using 2-D training images only. The lack of information is compensated by additional hypothesis about the symmetry of the 3-D simulation domain. In practice, the 3-D simulation domain is filled by a sequence of 2-D MPS simulations, performed alternatively along the directions where a 2-D training image is available. At each 2-D simulation step, all voxels simulated in the previous steps that cross the current 2-D simulation sub-domain are considered as conditioning data. By this, all 2-D sections in all directions are reconstructed in such a way that they are coherent with each other and also consistent with the structures in the training image. Here the 2-D training images are the facies distributions mapped for the two analogs along perpendicular vertical outcrops, and the 2-D MPS simulations are performed with the MPS simulation engine *impala*^52^.

## Data Records

For both analogs, we provide the results obtained with three MPS simulation settings. The first simulation setting consists of a model domain of the same size as the available mapped outcrops (320×200×140 voxels of 5 cm side for the Herten-analog; 280×70×58 voxels of 10 cm size for the Descalvado-analog), where the outcrops themselves are used as conditioning data to constrain the simulation. In the second setting, the same grid dimensions are used, but without considering the outcrops as conditioning data. For each of these two simulation settings, 100 equiprobable realizations are obtained by changing the simulation random seed. MPS uses the random numbers generated by a computer for the facies simulation and for the choice of the sequential simulation path. Using another random seed for each realization allows obtaining different stochastic simulations that are representative of the geological uncertainty. For the third simulation setting only one realization per analog is provided, that is a grid of size 1000×1000×140 for the Herten analog, and a grid of size 420×420×58 for the Descalvado analog.

## Technical Validation

The presented high resolution data sets exhibit several sources of uncertainty and inaccuracy, associated with each working step (Fig. 1). The sources may be grouped as those associated with mapping and facies assignment, those stemming from measurement inaccuracy, those related with parameter estimation and those originating from the geostatistical simulation procedure.

### Mapping, measurement and parameter estimation techniques

During mapping, facies types have been allocated by visual inspection of the outcrop wall and of outcrop photos. Even though the structural and textural properties, as well as the colours, were characteristic for the different facies, there is always a risk of misallocation. If possible, the mapped sequences have been validated by sedimentological analysis and comparison to other outcrop analogs^44^. This was crucial for obtaining sedimentologically plausible reconstructions. When several perspectives were available, such as for the Descalvado, consistency of the mapped facies mosaics was scrutinized at the intersections of perpendicular profiles.

The facies types were distinguished based on the principle that they present quasi-homogeneous units for the given scale of mapping (>5 cm). However, within facies types, there always remains a certain natural variability. In order to account for this variability and to arrive at robust parameter values, measurements of porosity, hydraulic conductivity, organic carbon and iron were all repeated several times with multiple samples per facies^11,31,32,34,40,53^. The detected value ranges are reflected for the listed parameters in Tables 2 and 3.

When no direct measurements were conducted, parameter values were estimated based on empirical calculations and by utilizing values reported for neighbouring sites. Empirical calculations were applied for hydraulic conductivity estimation. Since the applied standardized formula are only approximate, they were either utilized for plausibility checking (Herten^32^) or the derived values were successfully validated through in-situ flowmeter measurements (Descalvado^31^). No thermal measurements were conducted, and the given ranges are propagated uncertainties of the porosity. The chemofacies for the Herten analog are based on carbon measurements in two different adjacent gravel pits. Mean site-specific values of both pits are listed in Table 2 in order to highlight the spatial variability of the chemofacies types. However, this also shows that the regional hydrofacies-based classification may be suitable to also categorize chemo- and thermofacies at one site, but transferability to another site is in this case limited.

### Geostatistical simulation

The ensembles of 3-D realizations are validated using visual inspection and in terms of lithofacies proportions, total connectivity and intrinsic connectivity indicators^54,55^. The visual inspection allows evaluating the reliability and the geological realism of the simulated 3-D domains. A direct comparison of the proportions of the facies mapped in the field and the proportions of the lithofacies reproduced in the 3-D simulations is another important criterion to evaluate the simulations. A direct comparison of the connectivity indicators computed on the 2-D outcrops with the indicators computed on the 3-D simulations would be difficult to interpret. Therefore, while some preliminary tests are performed to compare the connectivity indicators of the 2-D datasets and 2-D MPS simulations, in the data repository we only attach the mean, the median and the standard deviation of the ensembles of 3-D s2Dcd simulations.

## Usage Notes

The ensembles of 3D realization of the Herten and the Descalvado analogs are uploaded in compressed format (zip) in the Open Access library PANGAEA (www.pangaea.de, (Data Citation 1)). For the first two simulation settings (conditional and unconditional simulations with domain sizes delimited by the datasets) 100 separate files (one for each realization) are provided for each analog and for each simulation setting. Only one file per analog is provided for the third simulation setting, which is for simulations of domains extended beyond the volumes delimited by the mapped outcrops. The files are provided in the Visualisation ToolKit (VTK, www.vtk.org) structured grid format, which can easily be read with the open source visualisation platform Paraview, freely downloadable (www.paraview.org), and which is described in detail in the VTK user’s guide^56^. The VTK files are provided in the ASCII format, with a header that contains a straightforward description of the size of the grid and its spacing (in meters). After the header, the facies codes are reported in a unique column starting from the values with lower *x* value, then lower *y* value, and then *z*. By simply changing the header, the files can be converted into the GSLIB file format (or simplified GeoEAS^57^), or read by mathematical libraries and scripting languages such as Python, Matlab or Mathematica in a straightforward manner. The README file attached to the data set includes a description of the workflow required for the conversion from VTK to other formats using a text editor, Python, Matlab/Octave or the bash shell. For each outcrop section, in addition to the VTK files, a VTI (VTK ImageData) file is provided to allow for direct visualization of the spatial variability of the hydraulic, thermal and chemical properties.

## Additional Information

**How to cite this article:** Bayer, P. *et al.* High resolution multi-facies realizations of sedimentary reservoir and aquifer analogs. *Sci. Data* 2:150033 doi: 10.1038/sdata.2015.33 (2015).

## Supplementary Material



## Figures and Tables

**Table 1:** Site conditions, geology and properties of Herten and Descalvado analogs.

	**Herten**	**Descalvado**
**Depositional environment**	fluvio-glacial	fluvial-aeolian
**Location**	Southern Germany (Rhine valley)	South-eastern Brazil
**Formation**	Würm late-glacial stage (Pleistocene)	Piramboia (Trias)
**Age**	ca. 15.000 y	250−145 my (GAS)
**Time of mapping**	1999	2011
**Size of sediment block**	16 m×7 m×10 m	28 m×5.8 m×7 m
**Volume of sediment block**	1120 m^3^	1136.8 m^3^
**Cross sections**	6, in parallel	3, in parallel; 2, perpendicular
**Maximum number of facies**	10	9
**Additional measurements**	Ground penetrating radar (GPR)	N/A

**Table 2:** Facies types and parameters of Herten analog.

**No.**	**Code**			**Lithofacies**	**Hydrofacies**		**Chemofacies**		**Thermofacies**	
	**Hydrofacies, Chemofacies, Thermofacies**	**%**	**Litho-facies**	**Description**	**Hydraulic conductivity (m/s)**	**Porosity (−)**	**Organic carbon content, f**_**OC,S**_ **(mg/g)**	**Organic carbon content, f**_**OC,H**_ **(mg/g)**	**Thermal conductivity, water saturated (W/m K)**	**Volumetric heat capacity, water saturated (MJ/m**^**3**^**K)**
0	Gcm	0.92	Gcm	poorly sorted, matrix supported gravel	2.5×10^−4^±2.1×10^−4^	0.17±0.07	0.64	0.33	3.23±0.47	2.42±0.15
1	cGcm	13.82	Gcm	poorly sorted, matrix supported gravel	2.3×10^−4^±2.1×10^−4^	0.15±0.01	0.64	0.33	3.37±0.07	2.38±0.02
2	sGcm	15.12	Gcm	poorly sorted, matrix supported gravel	6.1×10^−5^±5.9×10^−5^	0.13±0.04	0.39	0.33	3.53±0.29	2.34±0.08
3	Gcg,o	3.80	Gcg,a	alternating gravel	2.6×10^−2^±2.3×10^−2^	0.26±0.02	0.44	0.35	2.66±0.11	2.63±0.04
4	cGcg,o	26.21	Gcg,a	alternating gravel	1.3×10^−1^±7.4×10^−2^	0.26±0.02	0.42	0.35	2.64±0.11	2.63±0.04
5	sGcg,o	27.10	Gcg,a	alternating gravel	9.5×10^−2^±6.5×10^−3^	0.23±0.02	0.44	0.35	2.82±0.13	2.56±0.05
6	sGcm,b	0.35	Gcg,a	alternating gravel	4.3×10^−5^±1.8×10^−5^	0.22±0.02	0.46	0.33	2.92±0.14	2.53±0.05
7	fGcm,b	6.06	Gcg,a	alternating gravel	6.0×10^−7^±2.0×10^−7^	0.2±0.02	0.46	0.33	3.08±0.13	2.49±0.04
8	GS-x	5.26	GS-x	well sorted gravel (and coarse sand)	2.3×10^−3^±4.5×10^−4^	0.27±0.07	0.59	0.29	2.71±0.40	2.62±0.15
9	S-x	1.35	S-x	pure, well-sorted sand	1.4×10^−4^±5.0×10^−5^	0.36±0.04	0.43	0.19	2.38±0.21	2.78±0.08

**Table 3:** Facies types and parameters of Descalvado analog (note: organic carbon content was below detection limit of 0.04 mg/g).

**No.**	**Code**			**Lithofacies**	**Hydrofacies**		**Chemo-facies**	**Thermofacies**	
	**Hydrofacies, Chemofacies, Thermofacies**	**%**	**Litho-facies**	**Description**	**Hydraulic conductivity (m/s)**	**Porosity (−)**	**Fe(III) content (mg/g)**	**Thermal conductivity, water saturated (W/m K)**	**Volumetric heat capacity, water saturated (MJ/m**^**3**^**K)**
1	SGt,c	29.12	SGt	Trough-cross-bedded sand and gravel	3.0×10^−4^±9.9×10^−5^	0.32±0.04	0.17±0.05	2.66±0.24	2.67±0.09
2	SGt,m	1.72	SGt	Trough-cross-bedded sand and gravel	9.4×10^−5^±6.6×10^−5^	0.32±0.04	0.36±0.23	2.66±0.24	2.67±0.09
3	Sp,f	34.97	Sp	Planar-cross-bedded aeolian sand	1.6×10^−4^±1.7×10^−5^	0.25±0.05	1.86±0.22	3.12±0.35	2.51±0.11
4	Sh/Sp,m1	8.84	Sh/Sp	Horizontally laminated to planar cross-stratified sand	1.4×10^−3^±6.9×10^−5^	0.33±0.05	0.19±0.05	2.61±0.29	2.69±0.11
5	Sh/Sp,m2	1.33	Sh/Sp	Horizontally laminated to planar cross-stratified sand	7.8×10^−5^±3.1×10^−5^	0.33±0.05	0.08±0.03	2.61±0.35	2.69±0.13
6	St,m1	5.31	St	Trough-cross-bedded sand	6.0×10^−5^±2.9×10^−5^	0.29±0.04	1.79±0.65	2.85±0.25	2.60±0.09
7	St,m2	9.52	St	Trough-cross-bedded sand	2.5×10^−5^±1.3×10^−5^	0.29±0.05	5.08±0.59	2.85±0.32	2.60±0.11
8	St,f	9.16	St	Trough-cross-bedded sand	6.2×10^−6^±5.3×10^−6^	0.24±0.05	10.70±1.35	3.19±0.36	2.49±0.11
9	Fm	0.03	Fm	Massive clay intraclasts	7.8×10^−8^±4.2×10^−8^	0.29±0.03	57.47±15.09	1.90±0.10	3.00±0.05

## References

[d1] PANGAEA Bayer P. Comunian A. Höyng D. Mariethoz G. 2015 http://dx.doi.org/10.1594/PANGAEA.844167 10.1038/sdata.2015.33 PMC4493827 26175910

